# Cause of death during nasopharyngeal carcinoma survivorship: a population-based analysis

**DOI:** 10.3389/fonc.2023.1269118

**Published:** 2023-10-18

**Authors:** Jie Zhou, Zhenyu Jiang, Yunhao Li, Xuwen Shao, Haihong Liao

**Affiliations:** ^1^ Department of Oncology, Huzhou Central Hospital, Affiliated Central Hospital Huzhou University, Huzhou, China; ^2^ Department of Nephrology, The First People’s Hospital of Huzhou, First Affiliated Hospital of Huzhou University, Huzhou, China; ^3^ Department of Physical Examination Center, Zhejiang Xinda Hospital, Huzhou, China

**Keywords:** nasopharyngeal carcinoma, cause of death, SEER, survival, standardized mortality ratios (SMRs)

## Abstract

**Background:**

Recently, the survival rate of nasopharyngeal carcinoma (NPC) patients has improved greatly due to developments in NPC treatments. But cause-specific mortality in NPC patients remains unclear. This study aims to investigate the common causes of death in NPC patients.

**Methods:**

Eligible patients with NPC were included from the Surveillance, Epidemiology, and End Results (SEER) database. Standardized mortality ratios(SMRs) were calculated to compare death rates in NPC patients with those in the general population.

**Results:**

A total of 3475 patients with NPC were included, of whom 1696 patients died during the follow-up period. 52.83% of deaths were caused by NPC, followed by other cancers (28.13%) and non-cancer causes (18.46%). The proportion of patients who died of NPC decreased over survival time. Moreover, non-cancer causes of death increase from 12.94% to 51.22% over time after 10 years of diagnosis. Heart diseases was the most common non-cancer cause of death in NPC patients.

**Conclusions:**

Although NPC remains the leading cause of death after NPC diagnosis, other non-NPC causes of death represent an increased number of death in NPC patients. These findings support the involvement of multidisciplinary care for follow-up strategy in NPC patients.

## 1 Introduction

Nasopharyngeal carcinoma (NPC) is an epithelial carcinoma characterized by distinct geographical and ethnic distribution. Multiple risk factors contribute to the occurrence of NPC, including Epstein-Barr virus (EBV) infection, genetic predisposition and environmental factors (1, 2). Recently, there have been rapid evolution of the treatments in NPC patients. With the progress of radiotherapy techniques, optimization of chemotherapy strategies and breakthroughs of immune checkpoint inhibitors, the mortality of NPC patients has been substantially reduced (3–5). In long-term NPC survivors, understanding different causes of death is significant to develop individual follow-up strategies.

Previous studies have well described the causes of death from prostate cancer, breast cancer, small cell lung cancer and other cancers (6–10). But few existing studies have focused on the causes of death from NPC, especially for non-cancer reasons (11). Therefore, risk of death can be underestimated, and early intervention can not be carried out timely. In the current study, we endeavored to concentrate on each cause of death during NPC patients survivorship. We provided the analysis based on demographic-related and tumor-related characteristics and compared the risk of death from each cause with that of the general population.

## 2 Materials and methods

### 2.1 Data source

This was a retrospective cohort study. Data was collected from the Surveillance, Epidemiology, and End Results (SEER) 17 registries, November 2021 submission (2000–2019) for SMRs, which includes approximately 26.5% of the U.S. population.

### 2.2 Patients

**Figure 1:**
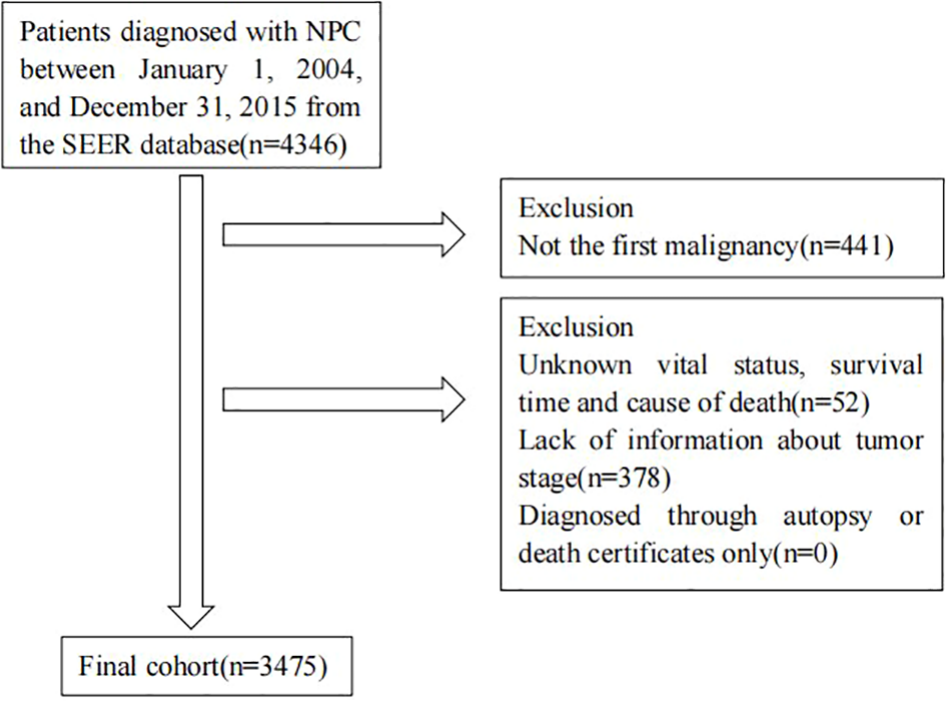
Flowchart of participant selection.

We identified all patients diagnosed with NPC as their first malignancy between 2004 and 2015 from SEER database. Patients diagnosed through autopsy or death certificates only were excluded. We also excluded patients with an unknown vital status, survival time, cause of death or staging information. **Figure 1** shows the flowchart of participant selection.

### 2.3 Study variables

Demographic and clinical information was extracted from SEER database, including sex, age, race, marital status, year of diagnosis, the 6th AJCC stage, histology type and treatment(surgery, radiotherapy or chemotherapy). Causes of death were mainly classified into cancer-related death and non-cancer death.

### 2.4 Outcome assessments

The number of all deaths after NPC diagnosis was calculated in different variables during all follow-up time and at each follow-up period. The cause of death record in SEER database was based on the International Statistical Classification of Diseases and Related Health Problems 10th Revision.

### 2.5 Statistical analysis

We computed standardized mortality ratios (SMRs), defined as the ratios of observed number of deaths in included NPC patients to expected number of deaths in the general population. Expected number of deaths was adjusted by age, sex, race and calendar year. We calculated the SMRs with 95% confidence intervals by SEER*Stat (version 8.4.0.1). All statistical tests were two-sided and a *p* value < 0.05 was considered to be statistically significant.

## 3 Results

### 3.1 Baseline characteristics

A total of 3475 patients diagnosed with NPC were included in our study. The number of male patients (72.26%) was 2.6 times higher than that of female patients (27.74%). Most patients (78.73%) were < 65 years old. The proportion of stage I-II patients (29.99%) and stage III patients (29.72%) was similar, while stage IV patients (40.29%) outnumbered. The majority of patients had keratinizing squamous cell histology type (43.71%) and non-keratinizing cell histology type (54.99%). Both radiotherapy and chemotherapy were common treatments for NPC patients.

During the follow-up, 1696 (48.81%) patients died. Of total deaths, 26.89% occurred within the first year of diagnosis, 50.18% occurred from 1 to 5 years, 18.10% occurred from 5 to 10 years, and 4.83% individuals survived longer than 10 years. In addition, 52.83% of death were caused by NPC, followed by other cancers (28.13%) and non-cancer causes (18.46%). Besides, NPC patients were at a higher risk dying from most other cancers. As for non-cancer causes, heart disease was the most common one (4.6%).

**Table 1** shows the baseline characteristics of patients with NPC included in our study. **Table 2** shows observed deaths and SMRs for causes of death after diagnosis of NPC. **Figure 2** shows causes of death after NPC diagnosis within each follow-up period.

**Table 1:** Baseline characteristics of patients with nasopharyngeal carcinoma.

Group	All Patients Diagnosed With NPC,No. (%)	Time of Death After Diagnosis,No. (%)				
		All Years	<1 year	1 to <5 years	5 to <10 years	≥10 years
All patients	3475 (100%)	1696 (100%)	456 (100%)	851 (100%)	307 (100%)	82 (100%)
Sex						
Male	2511 (72.26%)	1284 (75.71%)	334 (73.25%)	655 (76.97%)	231 (75.24%)	64 (78.05%)
Female	964 (27.74%)	412 (24.29%)	122 (26.75%)	196 (23.03%)	76 (24.76%)	18 (21.95%)
Age,years						
<65	2736 (78.73%)	1180 (69.58%)	265 (58.11%)	632 (74.27%)	221 (71.99%)	62 (75.61%)
≥65	739 (21.27%)	516 (30.42%)	191 (41.89%)	219 (25.73%)	86 (28.01%)	20 (24.39%)
Race						
White	1651 (47.51%)	906 (53.42%)	275 (60.31%)	414 (48.65%)	168 (54.72%)	49 (59.76%)
Black	411 (11.83%)	226 (13.33%)	66 (14.47%)	120 (14.10%)	33 (10.75%)	7 (8.54%)
Other	1413 (40.67%)	564 (33.25%)	115 (25.22%)	317 (37.25%)	106 (34.53%)	26 (31.71%)
Marital status						
Married	2035 (58.56%)	932 (54.95%)	227 (49.78%)	463 (54.41%)	190 (61.89%)	52 (63.41%)
Never married	785 (22.59%)	364 (21.46%)	103 (22.59%)	191 (22.44%)	57 (18.57%)	13 (15.85%)
Other	655 (18.85%)	400 (23.58%)	126 (27.63%)	197 (23.15%)	60 (19.54%)	17 (20.73%)
Year of diagnosis						
2004-2007	1027 (29.55%)	603 (35.55%)	139 (30.48%)	256 (30.08%)	134 (43.65%)	74 (90.24%)
2008-2011	1185 (34.10%)	601 (35.44%)	157 (34.43%)	299 (35.14%)	137 (44.63%)	8 (9.76%)
2012-2015	1263 (36.35%)	492 (29.01%)	160 (35.09%)	296 (34.78%)	36 (11.73%)	0 (0)
Stage						
I/II	1042 (29.99%)	374 (22.05%)	63 (13.82%)	168 (19.74%)	109 (35.50%)	34 (41.46%)
III	1033 (29.73%)	455 (26.83%)	106 (23.25%)	240 (28.20%)	91 (29.64%)	18 (21.95%)
IV	1400 (40.29%)	867 (51.12%)	287 (62.94%)	443 (52.06%)	107 (34.85%)	30 (36.59%)
Histology						
KSCC	1519 (43.71%)	920 (54.25%)	298 (65.35%)	432 (50.76%)	151 (49.19%)	39 (47.56%)
NKC	1911 (54.99%)	751 (44.28%)	153 (33.55%)	409 (48.06%)	147 (47.88%)	42 (51.22%)
BSCC	45 (1.29%)	25 (1.47%)	5 (1.10%)	10 (1.18%)	9 (2.93%)	1 (1.22%)
Surgery						
Yes	387 (11.14%)	160 (9.43%)	33 (7.24%)	86 (10.11%)	29 (9.45%)	12 (14.63%)
No/unknow	3088 (88.86%)	1536 (90.57%)	423 (92.76%)	765 (89.89%)	278 (90.55%)	70 (85.37%)
Radiation						
Yes	3079 (88.60%)	1403 (82.72%)	312 (68.42%)	731 (85.90%)	284 (92.51%)	76 (92.68%)
No/unknow	396 (11.40%)	293 (17.28%)	144 (31.58%)	120 (14.10%)	23 (7.49%)	6 (7.32%)
Chemotherapy						
Yes	2944 (84.72%)	1393 (82.13%)	318 (69.74%)	739 (86.84%)	265 (86.32%)	71 (86.59%)
No/unknow	531 (15.28%)	303 (17.87%)	138 (30.26%)	112 (13.16%)	42 (13.68%)	11 (13.41%)

NPC, nasopharyngeal carcinoma; KSCC, keratinizing squamous cell carcinoma; NKC, non-keratinizing carcinoma; BSCC, basaloid squamous cell carcinoma.

**Table 2:** Observed deaths and SMRs for causes of death after diagnosis of nasopharyngeal carcinoma.

	Timing of Death After Diagnosis									
	All years		<1 year		1 to <5 years		5 to <10 years		≥10 years	
	No. (%)	SMR (95% CI)	No. (%)	SMR (95% CI)	No. (%)	SMR (95% CI)	No. (%)	SMR (95% CI)	No. (%)	SMR (95% CI)
Cause of Death	1696 (100%)	7.83# (7.46-8.21)	456 (100%)	17.25# (15.7-18.91)	851 (100%)	8.81# (8.22-9.42)	307 (100%)	4.41# (3.93-4.93)	82 (100%)	3.44# (2.73-4.27)
All Malignant Cancers	1373 (80.96%)	23.30# (22.09-24.57)	396 (86.84%)	54.27# (49.05-59.89)	743 (87.31%)	28.05# (26.07-30.15)	195 (63.52%)	10.41# (9-11.98)	39 (47.56%)	6.09# (4.33-8.32)
Nasopharynx	896 (52.83%)	4,376.10# (4094.22-4672.28)	235 (51.54%)	9,246.20# (8101.73-10507.04)	525 (61.69%)	5,585.67# (5118.03-6084.56)	119 (38.76%)	1,859.63# (1540.55-2225.33)	17 (20.73%)	796.22# (463.83-1274.83)
Oropharynx	26 (1.53%)	202.78# (132.47-297.13)	14 (3.07%)	1,001.17# (547.35-1679.79)	10 (1.18%)	181.91# (87.23-334.54)	2 (0.65%)	46.28# (5.6-167.18)	0 (0)	0 (0-229.93)
Oral Cavity and Other Pharynx	80 (4.72%)	22.67# (17.97-28.21)	29 (6.36%)	38.05# (25.48-54.65)	38 (4.47%)	21.94# (15.53-30.11)	8 (2.61%)	8.34# (3.60-16.44)	5 (6.10%)	65.30# (21.20-152.38)
Digestive System	41 (2.42%)	2.29# (1.64-3.11)	8 (1.75%)	3.73# (1.61-7.35)	17 (2.00%)	2.13# (1.24-3.41)	12 (3.91%)	2.09# (1.08-3.64)	4 (4.88%)	1.99 (0.54-5.1)
Respiratory System	103 (6.07%)	6.22# (5.07-7.54)	28 (6.14%)	12.93# (8.59-18.69)	42 (4.94%)	5.54# (3.99-7.49)	25 (8.14%)	4.86# (3.15-7.17)	8 (9.76%)	4.76# (2.06-9.38)
Bones and Joints/Soft Tissue	5 (0.29%)	18.07# (5.87-42.17)	1 (0.22%)	28.94 (0.73-161.24)	1 (0.12%)	6.24 (0.16-34.79)	3 (0.98%)	36.58# (7.54-106.89)	0 (0)	0 (0-0)
Skin excluding Basal and Squamous	23 (1.36%)	21.69# (13.75-32.54)	11 (2.41%)	82.60# (41.23-147.79)	9 (1.06%)	18.82# (8.6-35.72)	3 (0.98%)	8.83# (1.82-25.81)	0 (0)	0 (0-33.73)
Breast/Genital/Urinary/Endocrine System	6 (0.35%)	13.60# (4.99-29.61)	0 (0)	0 (0-54.47)	3 (0.35%)	12.96# (2.67-37.86)	3 (0.98%)	21.16# (4.36-61.85)	0 (0)	0 (0-0)
Brain and Other Nervous System	8 (0.47%)	5.37# (2.32-10.58)	4 (0.88%)	22.73# (6.19-58.19)	4 (0.47%)	6.02# (1.64-15.42)	0 (0)	0 (0-7.64)	0 (0)	0 (0-22.16)
lymph/Blood	14 (0.83%)	9.74# (5.32-16.34)	3 (0.66%)	9.83# (2.03-28.73)	8 (0.94%)	8.36# (3.61-16.48)	2 (0.65%)	15.61# (1.89-56.38)	1 (1.22%)	20.96 (0.53-116.79)
Miscellaneous Malignant Cancer	171 (10.08%)	40.43# (34.6-46.97)	63 (13.82%)	119.70# (91.98-153.15)	86 (10.11%)	45.28# (36.22-55.92)	18 (5.86%)	13.43# (7.96-21.23)	4 (4.88%)	8.63# (2.35-22.1)
In situ, benign or unknown behavior neoplasm	10 (0.59%)	8.06# (3.87-14.82)	1 (0.22%)	6.65 (0.17-37.08)	4 (0.47%)	7.27# (1.98-18.6)	4 (1.30%)	9.97# (2.72-25.52)	1 (1.22%)	7.22 (0.18-40.23)
Noncancer	313 (18.46%)	9.25# (8.25-10.33)	59 (12.94%)	13.10# (9.97-16.89)	104 (12.22%)	6.16# (5.03-7.47)	108 (35.18%)	10.23# (8.39-12.35)	42 (51.22%)	22.04# (15.88-29.79)
Infections	26 (1.53%)	22.20# (14.50-32.52)	9 (1.97%)	44.26# (20.24-84.02)	8 (0.94%)	15.11# (6.52-29.77)	7 (2.28%)	27.08# (10.89-55.80)	2 (2.44%)	11.11# (1.35-40.14)
Diabetes Mellitus	6 (0.35%)	0.72 (0.26-1.56)	3 (0.66%)	3.05 (0.63-8.92)	1 (0.12%)	0.27 (0.01-1.52)	0 (0)	0 (0-1.36)	2 (2.44%)	2.03 (0.25-7.32)
Alzheimers (ICD-9 and 10 only)	6 (0.35%)	1.4 (0.51-3.04)	0 (0)	0 (0-33.16)	2 (0.24%)	1.07 (0.13-3.88)	3 (0.98%)	2.05 (0.42-6)	1 (1.22%)	2 (0.05-11.14)
Diseases of Heart	78 (4.60%)	1.52# (1.20-1.89)	16 (3.51%)	2.49# (1.42-4.04)	23 (2.70%)	1.00 (0.63-1.50)	28 (9.12%)	1.70# (1.13-2.46)	11 (13.41%)	1.98 (0.99-3.54)
Hypertension without Heart Disease	5 (0.29%)	1.99 (0.65-4.64)	0 (0)	0 (0-72.07)	1 (0.12%)	0.92 (0.02-5.13)	2 (0.65%)	2.39 (0.29-8.63)	2 (2.44%)	6.53 (0.79-23.60)
Cerebrovascular Diseases	21 (1.24%)	1.97# (1.22-3.01)	0 (0)	0 (0-14.81)	6 (0.71%)	1.26 (0.46-2.75)	11 (3.58%)	3.22# (1.61-5.77)	4 (4.88%)	3.37 (0.92-8.63)
Diseases of Arteries	4 (0.24%)	28.01# (7.63-71.71)	0 (0)	0 (0-45.12)	4 (0.47%)	65.50# (17.85-167.71)	0 (0)	0 (0-0)	0 (0)	0 (0-0)
Pneumonia and Influenza	17 (1.00%)	4.05# (2.36-6.49)	4 (0.88%)	7.65# (2.08-19.58)	8 (0.94%)	4.33# (1.87-8.53)	4 (1.30%)	2.91 (0.79-7.46)	1 (1.22%)	2.22 (0.06-12.37)
Chronic Obstructive Pulmonary Disease and Allied Cond	29 (1.71%)	2.61# (1.75-3.75)	3 (0.66%)	2.25 (0.46-6.58)	9 (1.06%)	1.84 (0.84-3.49)	13 (4.23%)	3.58# (1.91-6.13)	4 (4.88%)	3.19 (0.87-8.16)
Chronic Liver Disease and Cirrhosis	6 (0.35%)	1.33 (0.49-2.90)	0 (0)	0 (0-63.65)	4 (0.47%)	1.97 (0.54-5.04)	1 (0.33%)	0.69 (0.02-3.85)	1 (1.22%)	2.08 (0.05-11.57)
Nephritis/Nephrotic Syndrome/Nephrosis	1 (0.06%)	0.24 (0.01-1.34)	1 (0.22%)	1.99 (0.05-11.06)	0 (0)	0 (0-2.00)	0 (0)	0 (0-2.77)	0 (0)	0 (0-7.71)
Pregnancy/Childbirth/Puerperium	2 (0.12%)	100.92# (12.22-364.57)	1 (0.22%)	365.55# (9.25-2036.71)	1 (0.12%)	101.95# (2.58-568.02)	0 (0)	0 (0-640.84)	0 (0)	0 (0-2432.82)
Symptoms,signs and ill-defined conditions	5 (0.29%)	2.51 (0.82-5.87)	1 (0.22%)	3.85 (0.10-21.45)	2 (0.24%)	2.18 (0.26-7.88)	2 (0.65%)	3.23 (0.39-11.67)	0 (0)	0 (0-19.21)
Accidents and Adverse Effects	22 (1.30%)	2.15# (1.35-3.25)	5 (1.10%)	4.08# (1.32-9.52)	7 (0.82%)	1.52 (0.61-3.13)	8 (2.61%)	2.44# (1.05-4.80)	2 (2.44%)	1.78 (0.22-6.43)
Suicide and Self-Inflicted Injury	6 (0.35%)	1.76 (0.65-3.84)	4 (0.88%)	9.33# (2.54-23.90)	1 (0.12%)	0.63 (0.02-3.53)	1 (0.33%)	0.94 (0.02-5.26)	0 (0)	0 (0-10.95)
Homicide and Legal Intervention	3 (0.18%)	2.67 (0.55-7.80)	1 (0.22%)	6.80 (0.17-37.87)	2 (0.24%)	3.79 (0.46-13.70)	0 (0)	0 (0-11.09)	0 (0)	0 (0-31.33)
Other Cause of Death	76 (4.48%)	2.58# (2.03-3.23)	11 (2.41%)	3.26# (1.63-5.83)	25 (2.94%)	1.94# (1.25-2.86)	28 (9.12%)	2.87# (1.91-4.15)	12 (14.63%)	3.50# (1.81-6.12)

CI, confidence interval; SMR, standardized mortality ratio.

#p < 0.05.

**Figure 2:**
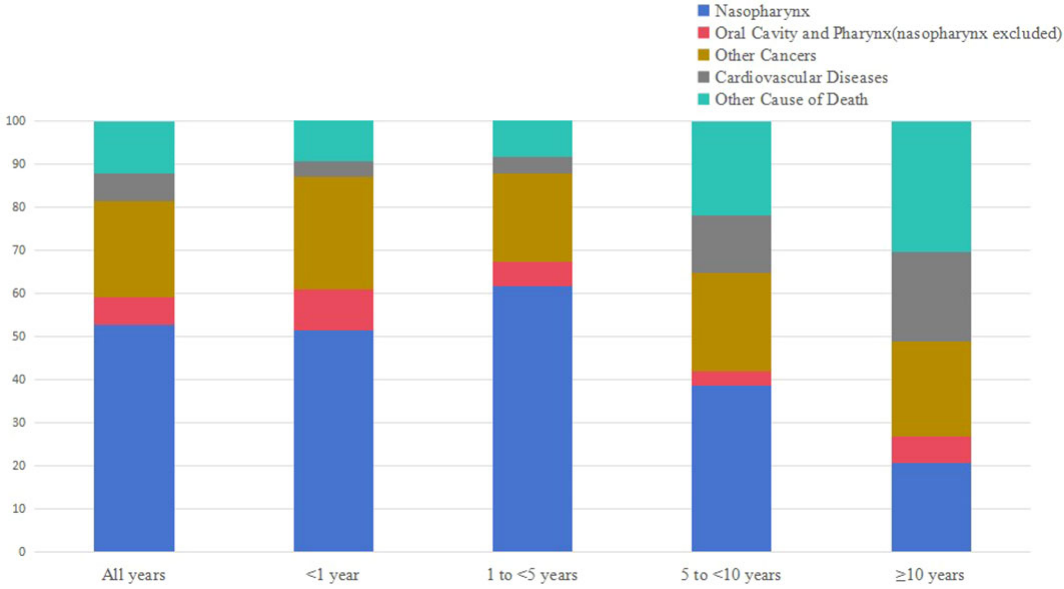
Causes of death after nasopharyngeal carcinoma diagnosis within each follow-up period.

### 3.2 Cause of death within 1 year following NPC diagnosis

Within 1 year after the diagnosis of NPC, a total of 456 death occurred. 51.54% died of NPC, 35.30% died of other cancers, and 12.94% died of non-cancer causes. Miscellaneous malignant cancer (13.82%) was the most common causes of other cancer deaths, followed by oral cavity and other pharynx cancers (6.36%). For non-cancer causes, the leading causes were heart diseases (3.51%), other cause of death (2.41%), and infections (1.97%), respectively. Besides, the risks of NPC patients dying from pregnancy/childbirth/puerperium (SMR 365.55#; 95%CI 9.25-2036.71), infections (SMR 44.26#; 95%CI 20.24-84.02), suicide and self-inflicted injury (SMR 9.33#; 95%CI 2.54-23.90), and pneumonia and influenza (SMR 7.65#; 95%CI 2.08-19.58) were 5 times higher than what expected in general population.

### 3.3 Cause of death within 1-5 years following NPC diagnosis

Within 1-5 years following NPC diagnosis, a total of 851 death occurred. NPC was the leading cause of death (61.69%), while other cancers (25.62%) and non-cancer causes (12.22%) accounting for the remaining proportion. Miscellaneous malignant cancer (10.11%) and heart diseases (2.70%) continued to be the most common causes of other cancer deaths and non-cancer cause of death. The SMR elevated to the highest level for pregnancy/childbirth/puerperium (SMR 101.95#; 95%CI 2.58-568.02), diseases of arteries (SMR 65.50#; 95%CI 17.85-167.71), and infections (SMR 15.11#; 95%CI 6.52-29.77) for non-cancer causes.

### 3.4 Cause of death within 5-10 years following NPC diagnosis

Within 5-10 years following NPC diagnosis, a total of 307 death occurred. 38.76% died of NPC, 24.76% died of other cancers, and 35.18% died of non-cancer causes. The respiratory system cancers (8.14%) turned to be the most common cause of other cancer death. Heart diseases (9.12%) remained to be the leading non-cancer cause of death. The mortality rate of infections was the highest in non-cancer causes of death (SMR 27.08#; 95%CI 10.89-55.80).

### 3.5 Cause of death after 10 years following NPC diagnosis

After 10 years of survival after NPC diagnosis, 82 patients died. 20.73% died of NPC, 26.83% died of other cancers, and 51.22% died of non-cancer causes. The proportion of respiratory system cancers(9.76%) was the highest one for patients died of other cancer causes. The frequency of NPC patients dying of heart diseases reached 13.41% among patients who survived more than 10 years. Patients with NPC also had a higher risk of infections related death (SMR 11.11#; 95%CI 1.35-40.14).

### 3.6 Subgroup analysis

Male and female NPC patients had a similar risk of cancer related death and non-cancer causes of death, but female patients (SMR 10434.51#; 95%CI 9150.85-11847.82) had a higher risk of death from NPC than male patients (SMR 3616.59#; 95%CI 3345.50-3903.80) (**Tables S1**, **2**). Patients aged < 65 years old had a higher risk of death than that of patients ≥ 65 years, no matter it was a cancer or non-cancer factor (**Tables S3**, **4**). The risk of all cause death in patients of other races (SMR 10.09#; 95%CI 9.28-10.96) was higher than white(SMR 6.95#; 95%CI 6.51-7.42) and black (SMR 7.45#; 95%CI 6.51-8.48) patients (**Tables S5**, **7**). Compared with patients who are married or in other marital statues, patients who have never been married had a higher risk of death, regardless of cancer or non-cancer causes (**Tables S8**-**10**). Patients diagnosed in recent years showed an increase in non-cancer causes of death (**Tables S11**-**13**). Patients with stage IV NPC had a higher risk of death from caner or non-cancer causes than patients with I-III NPC (**Tables S14**-**16**). The prognosis of patients with nasopharyngeal basaloid squamous cell carcinoma (BSCC) was the worst, while the prognosis of patients with nasopharyngeal non-keratinizing carcinoma (NKC) was the best (**Tables S17**-**19**). Patients who underwent surgery (SMR 14.77#; 95%CI 12.27-17.62) had a lower risk of cancer related death than those without surgery (SMR 24.71#; 95%CI 23.36-26.12) (**Tables S20**-**21**). All causes of death were lower in patients who received radiotherapy (SMR 7.06#; 95%CI 6.69-7.43) than those who did not (SMR 16.56#; 95%CI 14.72-18.57) (**Tables S22**-**23**). There was no significant difference in risk of death among patients given chemotherapy or not (**Tables S24**, **25**).

## 4 Discussion

The survival time of NPC patients is extended because of improved anti-tumor treatments. Several studies have explored the malignant causes of NPC-related mortality, but information on non-cancer causes of death remains limited in NPC survivors (12, 13). Using population-based data from the united states, our study detailed the causes of death in NPC patients. These results provide vital guidance for the health maintenance of NPC patients.

Our findings shown that the proportion of NPC-related death decreased gradually with the extension of survival time, while death due to non-cancer causes increased. Among NPC patients who survived more than 10 years, the incidence of non-cancer related death reached 51.22%.

The most common non-cancer related cause of death from NPC patients was heart disease during the whole follow-up periods after NPC diagnosis. Previous studies have indicated that cancer patients confronted a higher risk of cardiovascular death throughout their lives (14–16). This may be attributable to the adverse effects from anti-tumor treatments (17). Concurrent chemoradiation therapy is the mainstay treatment for NPC patients, leading to a progressive and dynamic cardiovascular autonomic dysfunction (18). Besides, immune checkpoint inhibitors (ICIs) have greatly improved the survival rate of NPC patients in recent years. Meanwhile, cardiac toxicities caused by ICIs should not be ignored (19, 20). Therefore, early interventions with cardiologists in these patients is suggested to provide individualized comprehensive care.

The frequency of other non-cancer related causes of death changed over time, with cerebrovascular diseases, infectious diseases, COPD and allied conditions becoming more frequent as time passed after NPC diagnosis. Radiotherapy for NPC patients may cause cerebrovascular diseases including transient ischemic attack and ischemic stroke, which can lead to severe disability (21). Besides, intracranial aneurysms are a rare complication of radiotherapy, but irradiated NPC patients had higher morbidity and mortality rates after aneurysm rupture and a higher angiographic recurrence rate after treatment (22). In addition, NPC patients had a higher risk of death from infectious diseases. This may be due to the use of chemotherapy, which is associated with the dysfunction of immune system. In our analysis, COPD was another common non-cancer cause of death. Smoking is a risk factor affecting the occurrence of NPC and related to higher NPC mortality (23–25). Tight association between NPC and COPD may be partly owing to shared risk factor of tobacco use.

Furthermore, in the present study, we found that NPC patients are more likely to develop second primary cancers such as respiratory, digestive and miscellaneous malignant cancers. Previous studies also have shown an increased risk for second primary cancers in NPC patients (26). Immune suppression, shared genetic factors and shared environmental risk factors might account for the associations (27). Besides, second cancer risk after definitive intensity-modulated radiotherapy (IMRT) was substantial in NPC patients (28). So it is advocated that NPC survivors should follow proper screening measures for other cancers.

The anatomical location and high sensitivity of NPC to radiotherapy make it the main treatment modality. Radical radiotherapy is the preferred treatment for early-stage NPC patients and the cornerstone of multidisciplinary treatment for locally advanced NPC patients. Lower risk of all causes of death was also found in NPC patients who received radiotherapy in our study. With the developments of irradiation techniques, the way of radiotherapy in NPC is constantly changing. Compared to conventional radiotherapy, IMRT has improved local control rate and been widespread adopted in clinical practice (29, 30). Besides, previous studies have suggested that IMRT was associated with decreased risk of radiation-induced injury (31, 32). To manage the errors in the positioning of patients or compensate for movements of organs, various methods of image guided radiotherapy (IGRT) are being developed. Meanwhile, intensity-modulated proton therapy (IMPT) has shown excellent locoregional control rate and significantly reduce complications in comparison with IMRT, and prospective studies are warranted (33).

Chemotherapy is associated with multiple adverse events depending on anti-tumor drugs. Cisplatin is commonly used to treat locally advanced and metastasis NPC patients. Cisplatin-based concurrent chemoradiotherapy has been identified as standard treatment for locally advanced NPC patients. But cisplatin-based chemotherapy is known to increase the adverse effects of radiotherapy (34). The main adverse events of cisplatin is nephrotoxicity, which limits the use of cisplatin. Apart from hydration regimens, several possible therapeutic targets preventing nephrotoxicity have been identified (35). Other multiple adverse effects such as gastrointestinal reactions, hematotoxicity, neurotoxicity may also influence the quality of life in second-line or higher treatment settings of NPC patients.

Reirradiation in recurrent NPC patients presents unique challenges with significant treatment-related toxicities, such as subcutaneous necrosis, large-vessel integrity, dysphagia and middle ear dysfunction. Hyperfractionated IMRT could improve overall survival and decrease the risk of severe late complications (36). In addition, with recent progress of endoscopic techniques, endoscopic resection of locally recurrent NPC have been reported in recent years. Local endoscopic resection achieved higher survival rates with fewer adverse events (37, 38). In our study, NPC patients who underwent surgery had a lower risk of cancer related death than those without surgery. We hypothesize that surgery could be recommended for recurrent NPC patients with operable tumors.

Several limitations should be acknowledged in our study. First, the study was retrospective and we tried our best to reduce bias. We designed strict screening criteria to reduce selection bias and used SMRs to control differences in age, sex and race to reduce confounding bias. Second, information about treatments and complications is incomplete in SEER database, which may influence survival durations and death patterns. Finally, NPC is particular prevalent in East and Southeast Asia, but most participants in our study were white. Whether our findings can be expended into other races needs to be further investigated. Despite these limitations, our study provides the most comprehensive assessments of causes of death in NPC patients.

## 5 Conclusions

In summary, deaths from non-NPC causes account for approximately 1/2 during follow-up after NPC diagnosis. Moreover, as survival time prolonged, the incidence of death from non-NPC causes increased. Heart diseases, infections diseases, COPD and allied conditions were the most common non-cancer causes of death in NPC patients. Therefore, we should not only pay attention to anti-tumor therapy, but also take notice of the occurrence of other risks to achieve better long-term outcomes in NPC patients.

## Data availability statement

The original contributions presented in the study are included in the article/**Supplementary Material**. Further inquiries can be directed to the corresponding author.

## Ethics statement

Ethical approval was not required for the study involving humans in accordance with the local legislation and institutional requirements. Written informed consent to participate in this study was not required from the participants or the participants’ legal guardians/next of kin in accordance with the national legislation and the institutional requirements.

## Author contributions

JZ: Conceptualization, Data curation, Formal Analysis, Methodology, Writing – original draft, Writing – review & editing. ZJ: Conceptualization, Data curation, Formal Analysis, Methodology, Writing – original draft, Writing – review & editing. YL: Data curation, Methodology, Writing – original draft, Writing – review & editing. XS: Data curation, Methodology, Writing – original draft, Writing – review & editing. HL: Data curation, Methodology, Supervision, Writing – original draft, Writing – review & editing.
